# Genome-Wide Analysis of the ATP-Binding Cassette (ABC) Transporter Family in *Zea mays* L. and Its Response to Heavy Metal Stresses

**DOI:** 10.3390/ijms23042109

**Published:** 2022-02-14

**Authors:** Zhaolai Guo, Xinqi Yuan, Linyang Li, Ming Zeng, Jie Yang, Hong Tang, Changqun Duan

**Affiliations:** 1 School of Ecology and Environmental Sciences, Yunnan University, Kunming 650091, China; 2 Yunnan Key Laboratory for Plateau Mountain Ecology and Restoration of Degraded Environments, Yunnan University, Kunming 650091, China; 18314522882@163.com (Z.G.); xinqiyuan2022@163.com (X.Y.); lilinyang@mail.ynu.edu.cn (L.L.); mingz@ynu.edu.cn (M.Z.); yangj0212@163.com (J.Y.); hongtang98@163.com (H.T.)

**Keywords:** *Zea mays* L., ABC transporter family, heavy metal, phylogenetic analysis, expression profile

## Abstract

The ATP-binding cassette (ABC) transporter family is one of the largest eukaryotic protein families. Its members play roles in numerous metabolic processes in plants by releasing energy for substrate transport across membranes through hydrolysis of ATP. Maize belongs to the monocotyledonous plant family, Gramineae, and is one of the most important food crops in the world. We constructed a phylogenetic tree with individual ABC genes from maize, rice, sorghum, *Arabidopsis*, and poplar. This revealed eight families, each containing ABC genes from both monocotyledonous and dicotyledonous plants, indicating that the amplification events of ABC gene families predate the divergence of plant monocotyledons. To further understand the functions of ABC genes in maize growth and development, we analyzed the expression patterns of maize ABC family genes in eight tissues and organs based on the transcriptome database on the Genevestigator website. We identified 133 ABC genes expressed in most of the eight tissues and organs examined, especially during root and leaf development. Furthermore, transcriptome analysis of *ZmABC* genes showed that exposure to metallic lead induced differential expression of many maize ABC genes, mainly including *ZmABC 012*, *013*, *015*, *031*, *040*, *043*, *065*, *078*, *080*, *085*, *088*, *102*, *107*, *111*, *130* and *131* genes, etc. These results indicated that *ZmABC* genes play an important role in the response to heavy metal stress. The comprehensive analysis of this study provides a foundation for further studies into the roles of ABC genes in maize.

## 1. Introduction

ATP-binding cassette (ABC) proteins are found in all organisms, from bacteria to humans [1]. Eukaryotic ABC proteins can be divided into eight major subfamilies (A_H) based on factors such as domain organization, the number of additional domains present, and whether the protein is half-size or full-size. Subfamily H genes are not present in plants [2]. ABC proteins are named based on the fact that they possess one or two ATP binding cassettes or nucleotide-binding folds (NBFs), which share 30~40% identity among family members [2]. Each NBF contains 200 amino acid residues and three unique sequence motifs [3]. Kang et al. [4] used a simple approach to name ABC transporter proteins, such as those encoding only nucleotide-binding domains (NBDs) or transmembrane domains (TMDs), an ABCI subfamily of bacterial types. They divided the Arabidopsis ABC transporter proteins into eight subfamilies, namely ABCA~ABCG and AGCI. These constitute one of the largest, most versatile and oldest super protein families found [5]. Pang et al. analyzed maize ABC transporters and provided general descriptions of their classifications, basic structures, evolution track analysis and expression profiles [6]. The members of the ABC family have been divided into three main groups: full-molecule ABC transport proteins, half-molecule ABC transport proteins and soluble transport proteins [7]. Among them, ABCB is currently the second largest subfamily, second only to the ABCG subfamily. Widely distributed in prokaryotes and eukaryotes, ABC transporter proteins achieve transmembrane transport of substrates by binding and hydrolyzing ATP to release energy, and the substrates they transport mainly include peptides, sugars, amino acids, metal ions, alkaloids, vitamins, inorganic molecules, organic molecules, glutathione and cell metabolites [2,5,7,8].

The ABC transporter factor family has been identified in many plant species. Previous studies have shown that *Arabidopsis thaliana* has 127 ABC genes [7]; *Oryza sativa* L. has 123 [9]; *Hordeum vulgare* has 131 ABC genes [10]; *Prunus mume* has 162 [11]; *Solanum lycopersicum* has 154 [12]; *Glycine max* has 261 [13]; *Leguminous Lotus* root has 89 [14]; and *Zea mays* L. has 130 [6]. Plant ABC genes have a variety of functions, such as the transport of hormones, lipids, metals, pathogens, antibiotics [15,16,17], secondary metabolites, and exogenous substances. They are also involved in the interaction between plants and pathogens and in the regulation of ion channels [3]. Therefore, ABC proteins can act as importers, exporters, receptors and channels [5]. *AtPGP1* is involved in growth hormone export and promotes hypocotyl and root growth [18]. The ABCB subfamily is associated with lipid transport in mammals and is involved in the transport of growth hormones, secondary metabolites and exogenous substances in plants [19]. ABCC subfamily proteins function in the detoxification and regulation of metal ions, and maize ABCC subfamily proteins play a role in the regulation of vesicles and seed phytate anthocyanin content [20]. ABCG subfamily proteins in plants are involved in resistance to pathogens, anti-microbial terpenoids and growth hormone herbicides, and *AtABCG11* and *AtABCG12* in Arabidopsis are involved in the secretion of plant surface waxes [21] ABCH-like subfamily proteins have been identified in animals and are notably absent in plants. ABCI subfamily proteins contain mainly ABC proteins similar to prokaryotic transporter proteins; however, the functions of this class of proteins have not been investigated and need to be explored further [2].

Heavy metals have an atomic number greater than 20. There are approximately 40 heavy metals, including cadmium (Cd), chromium (Cr), mercury (Hg), lead (Pb), copper (Cu), zinc (Zn), silver (Ag) and tin (Sn) [22]. As a result of human activities, an increasing number of global regions are experiencing heavy metal levels above the normal range, leading to the deterioration of environmental quality and ecosystem health. Heavy metals pose a long-term threat to the natural environment and human survival because of their non-degradable nature, long-term presence and invisibility in the environment [23]. Within cells, heavy metals affect photosynthesis, respiration, mineral nutrition, enzyme reactions and many other physiological processes [24]. The excessive accumulation of heavy metals in plant tissues can reduce root length, plant biomass, seed germination and chlorophyll synthesis [25]. The inhibition of root growth, especially at the tip, is susceptible to certain heavy metals such as Al, Cu, Cd and Hg [26]. The effects of heavy metals on plants are multifaceted, mainly including plant growth, plant dry weight, net photosynthesis, stomatal conductance, etc. [27]. Such studies are important for analyzing plant mechanisms of physiological and ecological adaptation after short-term exposure to Pb and Cd. However, there is a paucity of research on persistent heavy metal stress. There is a need to understand and appreciate the dynamics of plant adaptation and evolutionary mechanisms in response to long-term persistent heavy metal pollution.

Maize is the largest and most widely distributed food crop in China, and its seeds and stalks are a source of feed for most animals and a raw material for agricultural processing. Its quality is closely related to the health of the nation. The United States leads the world with about half of the world’s annual corn production, then China, followed by Brazil, Mexico, and Argentina [28]. Abiotic stresses, such as drought, salinity, low temperature, and heavy metals, adversely affect plant growth and are important limiting factors of crop productivity [29]. Although Pang et al. and co-authors have reported the genes of the maize ABC transporter family [6], the scientific community’s systematic understanding of maize ABC genes is still limited, and the study of its response to environmental stress is unknown. Therefore, on the basis of the genome-wide analysis of ABC genes, this study highlights the response of ABC genes to heavy metals. We found that many of *ZmABC* genes (e.g., *ZmABC 040* and *ZmABC088*) from maize B were significantly up-regulated by exposure to lead, which may play important roles in maize tolerance to metals. This study may provide basic data for a comprehensive understanding of lead biotoxicity and the roles of maize ABC genes in resistance to lead.

## 2. Results

### 2.1. Identification of ABC Family Genes in Zea mays L.

**Figure 1:**
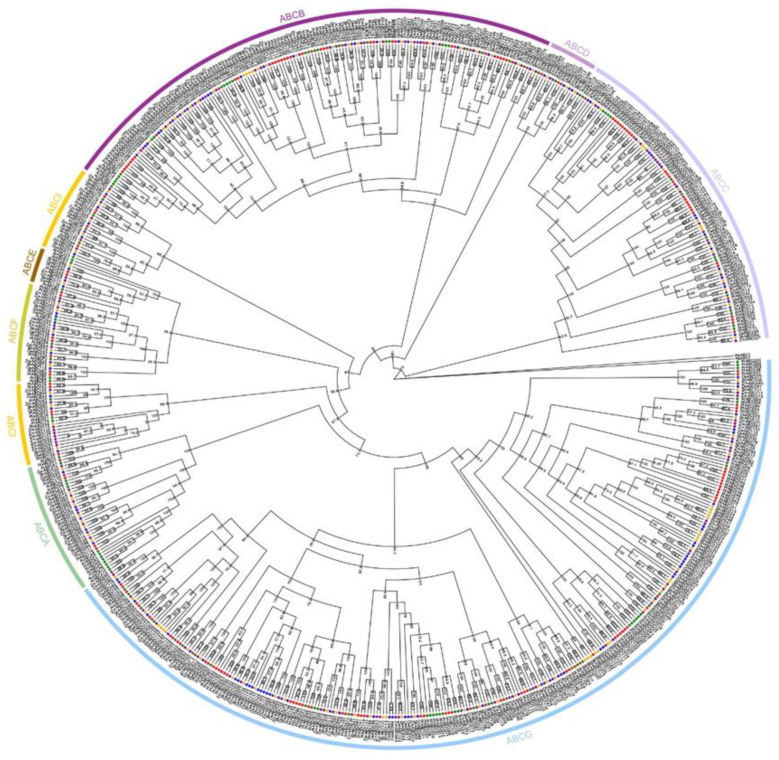
Evolutionary analysis of ABC family genes in maize cultivar B84 and other four different crops: Zm: *Zea mays* L.; Os: *Oryza sativa* L.; Sb: *Sorghum bicolor* (L.) Moench; At: Arabidopsis; Ptr: PopulusL.

The release of the complete maize genome allowed the genome-wide identification of genes. To investigate the evolutionary relationships of ABC genes, we constructed a phylogenetic tree using the amino acid sequences from the monocotyledonous plants, maize, rice and sorghum and the dicotyledonous plants, *Arabidopsis thaliana*, and poplar (Figure 1). The evolutionary tree included the following ABC genes: 133 in maize, 126 in rice, 133 in sorghum, 118 in *Arabidopsis* and 172 in poplar. These genes can be divided into eight distinct evolutionary branches. The ABCB and ABCG gene families have the most members, indicating that they play important roles in adaptation to the environment. ABCI family genes, which are rarely found in plants, were also identified.

The length of the ZmABC proteins ranged from 164 (ZmABC021) to 1627 (ZmABC027) amino acids, molecular weights ranged from 18.12 (ZmABC021) to 183.13 (ZmABC027) kDa, and the theoretical isoelectric point values ranged from 1.18 (ZmABC110) to 10.06 (ZmABC115). The c average hydrophobicity values ranged from −23.942 (ZmABC029) to 31.939 (ZmABC119), indicating that most ZmABC proteins were hydrophobic, except for ZmABC004, -014, -024, -027, -029, -031, -038, -039, -047, -049, -050, -052, -054, -061, -063, -069, -074, -079, -080, -083, -084, -090, -091, -098, -100, -101, -110, -113, -119, -122, -124, -128 and -13 (Table S1).

### 2.2. Conserved Domain and Motif Analysis 

**Figure 2:**
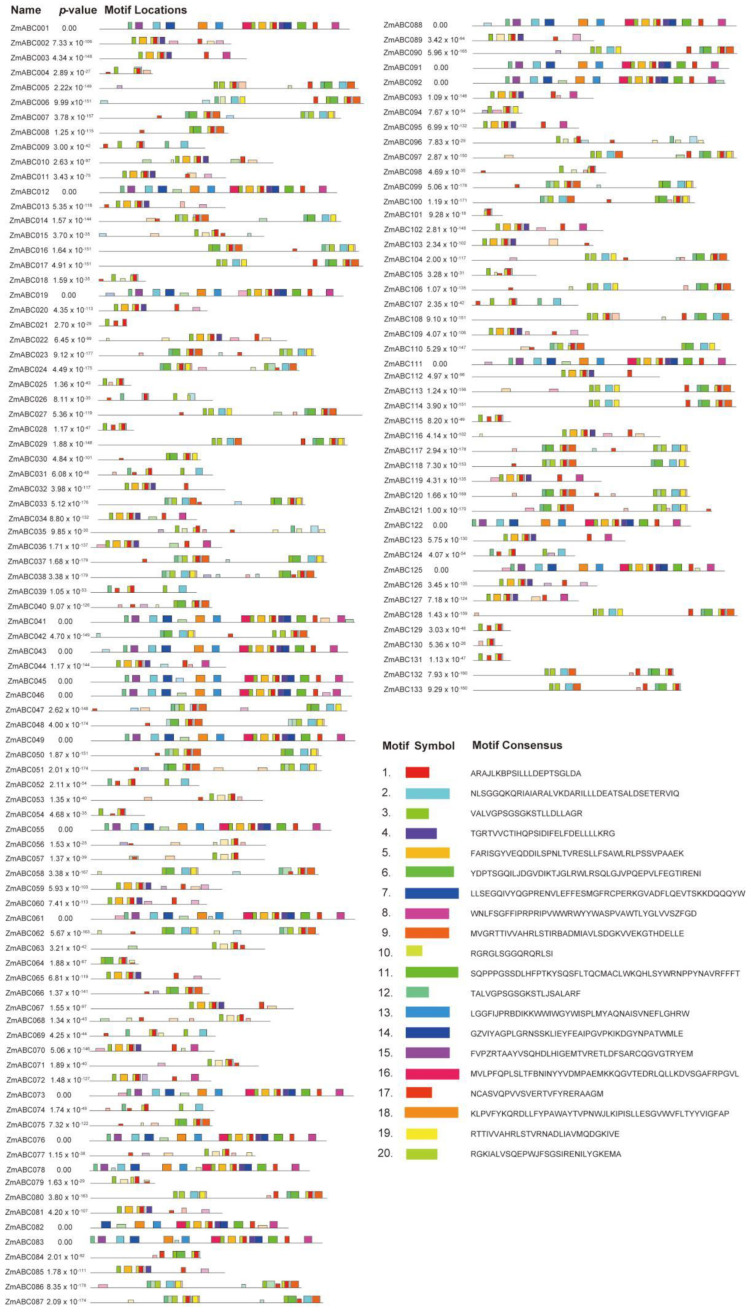
Genomic structure and motif composition of ZmABCs: the conserved motifs in ZmABCs proteins were identified using MEME. Each motif is represented with a specific color.

To better reveal the diversification among maize ABC genes, their conserved motifs were analyzed. The MEME online tool was used to predict the conserved motif compositions of the ZmABCs (Figure 2). The number of motifs varied from 3 to 19. Motif 1, the ABC domain, was identified in all ZmABCs. In addition, other motifs were only present in members of specific subclades, indicating that they have subclade-specific functions.

### 2.3. Analysis of Cis-Acting Elements in the Promoter Region of Maize ABC Family Genes

**Figure 3:**
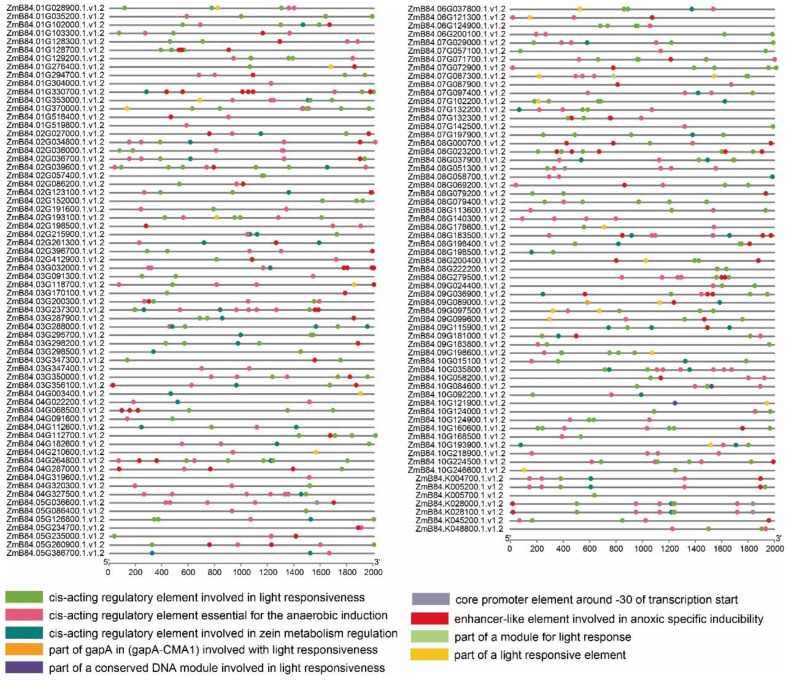
The cis-acting elements in the upstream promoter region of the maize ABC gene.

To better understand the function of maize ABC family genes, we analyzed the cis-acting elements in the 2000 bp promoter region of each gene (Figure 3). In addition to the essential cis-acting elements related to transcription and light response, three classes of cis-acting elements were identified. We found elements involved in stress responses, mainly including light responses, anaerobic sensing and regulation of protein metabolism. These results indicate that maize ABC genes play an important role in both plant growth and development as well as in adversity stress.

### 2.4. Analysis of Maize ABC Family Gene Expression Patterns in Different Tissues and Organs 

**Figure 4:**
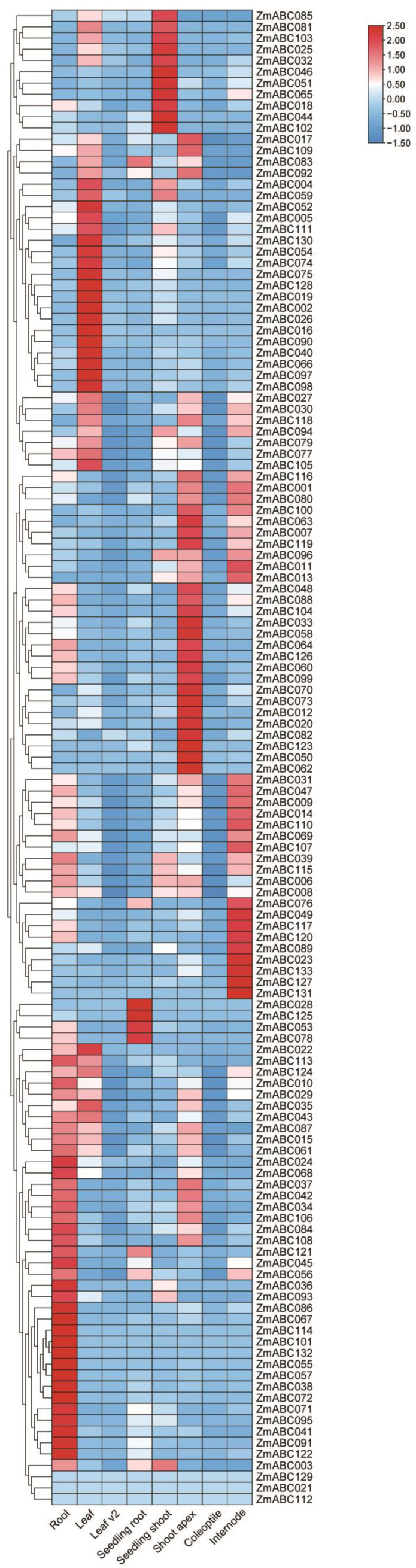
Using transcriptome data to obtain the expression levels of ABC family genes of maize cultivar B84 at different developmental stages. In the figure, the expression level of each gene in all tissues and organs tested is set to 100%, and the shade of the color represents the expression percentage.

To further understand the spatial expression patterns of maize ABC family genes, we used information archived in the transcriptome database of the HMMER 3.3.2 website. We analyzed the ABC family genes in eight different tissues and developmental stages of maize cultivar B84 (root, leaf, leaf v2, seedling root, seedling shoot, shoot apex, coleoptile and internode). As shown in Figure 4, 133 ABC genes were expressed in most of the eight different developmental stages. *ZmABC129*, *021* and *112* were expressed at the same level in all eight sites, indicating that the functions of these three genes may be the same at different sites. Most of the ZmABC genes were constitutively expressed and were expressed at all eight sites. No genes were highly expressed in leaf v2. These results indicate that ABC family genes are involved in regulating different developmental processes of maize. 

### 2.5. Analysis of ABC Gene Expression in Maize of Generation Pre-Adapted to Heavy Metal

Gene expression patterns correlate with function [30]. To explore the possible functions of ZmABC genes in maize, we grew maize on lead-contaminated soil for up to 20 generations and examined the transcriptomes of individuals from the 5th, 15th and 19th generations.

**Figure 5:**
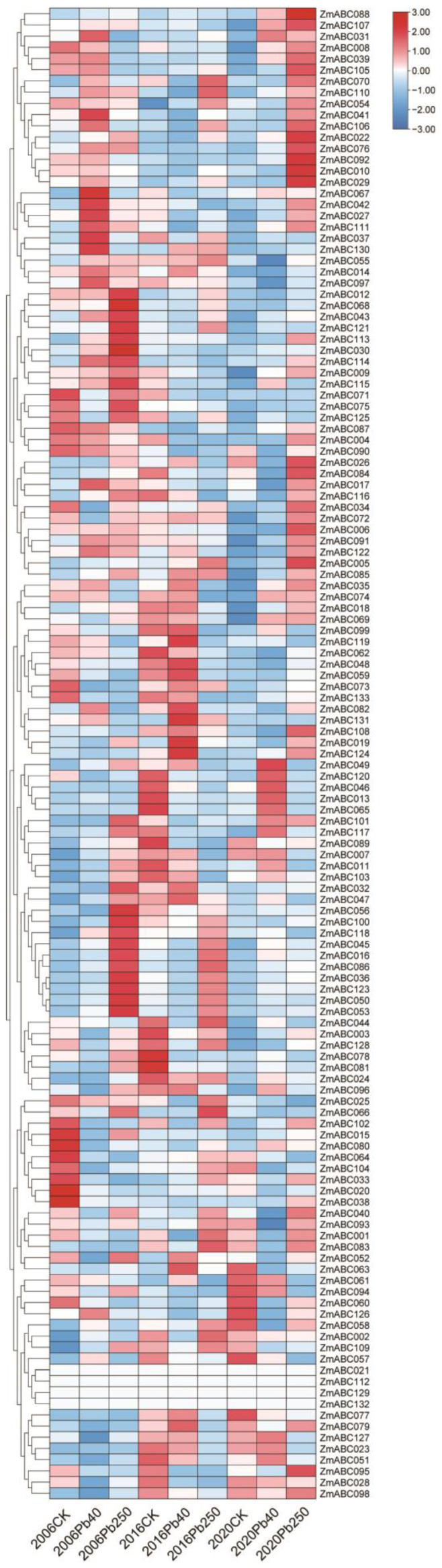
Expression analysis of ZmABC genes of maize cultivar B after treatment with Pb. 2006ck: 5th generation seeds under control check; 2006Pb40: 5th generation seeds under Pb40 mg/kg; 2006Pb250: 5th generation seeds under Pb250 mg/kg; 2016ck: 15th generation seeds under control check; 2016Pb40: 15th generation seeds under Pb40 mg/kg; 2016Pb250: 15th generation seeds under Pb250 mg/kg; 2020ck: 19th generation seeds under control check; 2020Pb40: 19th generation seeds under Pb40 mg/kg; 2020Pb250: 19th generation seeds under Pb250 mg/kg.

As shown in Figure 5, the expression of *ZmABC 021*, *112*, *129* and *132* genes was not observed in different generations and different concentrations of lead treatment. However, the differential expression of multiple genes was detected, such as *ZmABC 012*, *080* and *130* genes etc. In the 5th generation, exposure to lead mainly induced up-regulation of *ZmABC 012*, *043* and *111* genes compared with the control group and mainly induced down-regulation of *ZmABC 015*, *080* and *102* genes; in the 15th generation, exposure to lead mainly induced up-regulation of *ZmABC 085*, *130* and *131* genes compared with the control group and mainly induced down-regulation of *ZmABC 013*, *065* and *078* genes; in the 19th generation, exposure to lead mainly induced up-regulation of *ZmABC 031*, *040*, *088* and *107* genes compared with the control group and mainly induced down-regulation of *ZmABC 057*, *063* and *077* genes. These findings indicate that many *ZmABC* genes may play an important role in the response to persistent heavy metal stress.

## 3. Discussion

ABC transporter proteins are a group of transmembrane transport proteins commonly found in prokaryotes and eukaryotes that have a large and diverse range of functions. In plants, ABC transporter proteins are involved in maintaining cellular osmotic homeostasis, toxin enrichment and efflux and stomatal movement in response to various stresses and are essential for normal seed germination and lateral root development [31,32]. Phylogenetic trees were constructed using 118 ABCs of Arabidopsis, 126 of rice, 133 of sorghum, 172 of poplar, and 133 of maize (Figure 1). The results showed the 682 ABC genes from the five species scattered in each branch, indicating that the ABC gene family amplification event predates the differentiation of plant monodic types. 

Analysis of the transcriptome data showed that one or more ABC genes were expressed in each of the eight different maize tissues and organs, indicating that maize ABC genes are involved in several different growth and development processes, especially in leaf and root development (Figure 4). Heavy metals can adversely affect plants, and plants adapt to their environment by changing. To determine the expression of ZmABC genes and to understand the role of *ZmABC* genes in responses to heavy metals, we studied the transcriptome data using Pb 40 mg/kg and Pb 250 mg/kg treatments. The expression levels of *ZmABC* genes were affected by different heavy metal treatments (Figure 5). This indicates that ZmABC proteins may be involved in heavy metal responses and supports their role in the regulation of plant growth and development. The mechanisms by which *ZmABC* genes participate in heavy metal responses need to be further studied. Maize branch and root growth are reduced when grown on cadmium-contaminated soil [33,34]. Soil Pb contamination also reduces germination, inhibits growth, reduces plant biomass and reduces plant protein content in maize [34,35].

The function of ABC genes in Arabidopsis has been studied in relative detail, and the function of some ABC genes in maize can be inferred from the fact that evolutionarily related genes have similar functions. For example, *AtABCC1/AtMRP1* and *AtAB-CC2/AtMRP2* are a very important class of PCs that localized to vesicle membranes and mediate cell tolerance to cadmium and mercury [36,37], whereas *AtMRP6* detoxifies the heavy metal cadmium [38]. The expression of *AtABCG40/AtPDR12* is up-regulated under Pb stress. *AtABCG40* can transport lead or lead derivatives out of the cytoplasm, thereby enhancing the tolerance of Arabidopsis to lead [39]. At the same time, *AtABCG36/PDR8* detoxifies and regulates growth hormone [40]. However, few reports have investigated the function of ABC genes under heavy metal stress. Therefore, in this study, we systematically investigated the expression pattern of ABC genes in maize roots using RNA-Seq data from maize seedling roots of different generations and under different heavy metal stresses. The results of this study show that *ZmABC040* and *ZmABC088* were identified as possible candidates for lead resistance that merit further study. Plants regulate their growth under heavy metal contamination by changing the resource allocation of different organs to better adapt to survive [41,42]. The adaptation of plants to their environment is achieved through genetic regulation. This study provides a reference for future studies of the function of ABC genes under heavy metal stress.

## 4. Conclusions

We performed a genome-wide analysis of the ABC gene family in maize and revealed the different expression of *ZmABC* genes in eight different families. Meanwhile, expression analysis under long-term sustained stress showed that exposure to metallic lead induced differential expression of many maize ABC genes, mainly including *ZmABC 012*, *031*, *040*, *043*, *065*, *088* and *107* genes etc. These results indicated that *ZmABC* genes may play important roles under heavy metal stress. The results of this study lay the foundation for further studies on the regulations of ABC gene family members in growth and development and in response to heavy metals.

## 5. Materials and Methods

### 5.1. Plant Material

Maize cultivar B was cultivated in a 1:1 (*w*/*w*) soil: sand mixture. Outside, the plants were maintained at 16–24 °C in 60–80% relative humidity. The maize used in this study was obtained from the lead–zinc mining area in Lanping County, Yunnan Province, China. The maize variety is Maize B, which has experienced long-term heavy metal pollution.

Maize was planted in soil with Pb 40 mg/kg and Pb 250 mg/kg. CK refers to the treatment group without the addition of heavy metals (as the control group). Samples were quickly frozen in liquid nitrogen and stored at −80 °C until use.

### 5.2. Phylogenetic Tree Construction of ABC Genes from Maize, Rice, Sorghum, Arabidopsis and Poplar 

Genomic data were downloaded from Phytozome 13 (https://phytozome-next.jgi.doe.gov/, accessed on 9 October 2021) for Arabidopsis, rice, poplar, sorghum and maize cultivar B84. Sequences were found based on PF00005 seed files using HMMER v3.3.2 software. The obtained ABC genes were submitted to the pfam database (http://pfam.xfam.org, accessed on 10 October 2021) for structural domain prediction, retaining sequences containing the ABC_tran structural domain. MAFFT v7.487 was used for multiple sequence comparison. TrimAl v1.4.1 was used to trim the multiple sequence results. IQ-TREE 2.1.4-beta was used to construct ML trees with the model Q.plant +R10. Other parameters used were -bb 2000, -alrt 2000. EvolView (https://evolgenius.info/evolviewv2/#mytrees/JIEDAN/ABCGENE, accessed on 11 October 2021) was used to bdepict the evolutionary tree. Variety B84 maize data were downloaded from the plant gene database phytozome13 (https://phytozome-next.jgi.doe.gov/, accessed on 9 October 2021).

### 5.3. Conserved Motif Analysis

The conserved motifs of ZmABC proteins were analyzed using Multiple Em for Motif Elucidation (http://meme-suite.org/index.html (accessed on 23 November 2021)). The maximum number of motifs to be identified was set to 20. In addition, every sequence that occurred at least once was used [43].

### 5.4. Sequence Characterization

LaserGene7 was used to further analyze the identified ZmABCs, including analysis of coding sequence lengths, protein sizes, protein molecular weights, isoelectric points and averages of hydropathicity [43].

### 5.5. Spatiotemporal Expression Analysis of ZmABC Genes Using Transcriptome Data

Transcriptome data from different tissues of maize cultivar B84 were downloaded from NCBI. The data were filtered using trimmomatic v0.39 with parameters LEADING:20, TRAILING:20, SLIDINGWINDOW:4:20, and MINLEN: 50. Ribosomal RNA was removed using sortmerna v4.3.4. R was then used with Salmon 1.5.2 to calculate expression. Heat maps were produced using TBtools.

### 5.6. Analysis of Cis-Acting Elements in the Promoter Region

TBtools v.1_098665 was used to obtain 2000 bp of upstream coding sequence from maize ABC genes. Sequences were submitted to the PlantCare website for cis-acting elements (http://bioinformatics.psb.ugent.be/webtools/plantcare/html/, accessed on 13 October 2021), and then TBtools was used for visual analysis [44].

### 5.7. Transcriptiome Data

The transcriptome data for the different generations were obtained in our own experiments and are not currently uploaded to a database. Briefly, first, total RNA from maize B roots was extracted using the reagent test kit (Biospin, Billerica, MA, USA) according to the manufacturer’s instructions. Next, the concentration and integrity of RNA were analyzed by an Agilent 5400 spectrofluorometer (Agilent Technologies, Santa Clara, CA, USA). After that, transcriptome sequencing analysis was performed on the Illumina HiSeq platform (Illumina, San Diego, CA, USA). This research was supported by Wuhan Metware Biotechnology Co., Ltd. (Wuhan, China). Finally, DESeq2 v1.22.1 was used to analyze the differential expression between two groups, and the *p*-value was corrected using the Benjamini and Hochberg method. The corrected *p*-value and |log2foldchange| were used as the threshold for significant difference. A heatmap was constructed using Genesis.

## Data Availability

Not applicable.

## Supplementary Materials

The following supporting information can be downloaded at: https://www.mdpi.com/article/10.3390/ijms23042109/s1.
