# Supplementary material for: Genome-Wide Analysis of the ATP-Binding Cassette (ABC) Transporter Family in Zea mays L. and Its Response to Heavy Metal Stresses
Source: Int J Mol Sci. 2022 Feb 14;23(4):2109. doi: 10.3390/ijms23042109 (PMC8879807; doi:10.3390/ijms23042109)
Supplement: Supplementary file 1 [file ijms-23-02109-s001.zip › ijms-1556309-supplementary.pdf]

Supporting Information for

Genome-Wide Analysis of the ATP-Binding Cassette (ABC) Transporter Family in *Zea mays* L. and its Response to Heavy Metal Stresses

Zhaolai Guo[1,2], Xinqi Yuan[1,2], Linyang Li[1,2], Ming Zeng[1,2], Jie Yang[1,2], Hong Tang[1,2] and Changqun Duan[1,2*]

[1]School of Ecology and Environmental Sciences, Yunnan University, Kunming, 650091, People's Republic of China.

[2]Key Laboratory for Plateau Mountain Ecology and Restoration of Degraded Environments, Yunnan University, Kunming 650091, People's Republic of China.

*****Correspondence: chqduan@ynu.edu.cn; E-mail: chqduan@ynu.edu.cn (C D); Tel.: +86 871 65032753

Figures and Tables

Table S1. Identification and characteristics of ABC genes in Maize.

Gene name	Accession Number	CDS (bp)	Protein Size(aa)	MW (kD)	PI	GRAVY
ZmABC001	ZmB84.01G519800.1.v1.2	4290	1429	161.23	7.12	0.930
ZmABC002	ZmB84.01G035200.1.v1.2	2259	752	82.28	8.95	12.437
ZmABC003	ZmB84.01G103300.1.v1.2	2526	841	92.47	9.16	13.995
ZmABC004	ZmB84.01G128700.1.v1.2	912	303	32.36	5.48	-6.082
ZmABC005	ZmB84.01G276400.1.v1.2	4443	1480	163.24	7.94	10.125
ZmABC006	ZmB84.01G028900.1.v1.2	4533	1510	166.79	8.12	12.812
ZmABC007	ZmB84.01G304000.1.v1.2	4140	1379	148.11	8.84	18.809
ZmABC008	ZmB84.01G128300.1.v1.2	2214	737	80.72	9.07	13.571
ZmABC009	ZmB84.01G129200.1.v1.2	1815	604	68.26	7.65	3.174
ZmABC010	ZmB84.01G370000.1.v1.2	2979	992	109.60	8.20	14.100
ZmABC011	ZmB84.01G518400.1.v1.2	2169	722	79.66	8.44	8.44
ZmABC012	ZmB84.01G294700.1.v1.2	4074	1357	153.81	8.00	9.325
ZmABC013	ZmB84.01G353000.1.v1.2	2157	718	78.61	8.80	11.178
ZmABC014	ZmB84.01G102000.1.v1.2	4242	1413	155.96	6.16	-16.367
ZmABC015	ZmB84.01G330700.1.v1.2	2889	962	107.29	7.36	2.207
ZmABC016	ZmB84.02G198500.1.v1.2	4548	1515	162.21	9.17	24.516
ZmABC017	ZmB84.02G057400.1.v1.2	4623	1540	172.91	8.10	13.540
ZmABC018	ZmB84.02G379900.1.v1.2	822	273	30.14	8.07	2.621
ZmABC019	ZmB84.02G123100.1.v1.2	4278	1425	159.71	7.61	4.755
ZmABC020	ZmB84.02G215900.1.v1.2	1899	632	67.000	9.52	20.935
ZmABC021	ZmB84.02G036000.1.v1.2	495	164	18.12	9.49	4.225
ZmABC022	ZmB84.02G191600.1.v1.2	3291	1096	121.93	8.54	24.244
ZmABC023	ZmB84.02G027000.1.v1.2	3801	1266	137.24	8.06	6.305
ZmABC024	ZmB84.02G261300.1.v1.2	3720	1239	133.47	6.76	-1.674
ZmABC025	ZmB84.02G034800.1.v1.2	618	205	22.86	9.54	4.907
ZmABC026	ZmB84.02G396700.1.v1.2	2118	705	79.10	7.94	3.037
ZmABC027	ZmB84.02G039600.1.v1.2	4884	1627	183.13	6.76	-2.285
ZmABC028	ZmB84.02G036700.1.v1.2	663	220	24.81	9.76	6.905
ZmABC029	ZmB84.02G193100.1.v1.2	4611	1536	168.34	5.69	-23.942
ZmABC030	ZmB84.02G152000.1.v1.2	1902	633	68.77	9.03	12.245
ZmABC031	ZmB84.02G412900.1.v1.2	2124	707	77.47	6.02	-9.419
ZmABC032	ZmB84.02G086200.1.v1.2	2349	782	85.53	9.07	16.105
ZmABC033	ZmB84.03G298500.1.v1.2	3831	1276	139.10	7.49	3.682
ZmABC034	ZmB84.03G350000.1.v1.2	1830	609	65.20	9.53	15.914
ZmABC035	ZmB84.03G174300.1.v1.2	3972	1323	147.75	9.01	25.617
ZmABC036	ZmB84.03G295700.1.v1.2	2217	738	81.33	8.79	10.964
ZmABC037	ZmB84.03G091300.1.v1.2	3990	1329	143.17	8.59	10.184
ZmABC038	ZmB84.03G298200.1.v1.2	3822	1273	137.61	6.06	-9.614
ZmABC039	ZmB84.03G118700.1.v1.2	1788	595	66.23	6.52	-4.237

ZmABC040	ZmB84.03G200300.1.v1.2	2052	683	74.97	9.18	13.089
ZmABC041	ZmB84.03G347300.1.v1.2	4464	1487	166.73	8.05	7.390
ZmABC042	ZmB84.03G288000.1.v1.2	3696	1231	134.38	8.46	12.307
ZmABC043	ZmB84.03G347400.1.v1.2	4347	1448	163.52	7.17	1.639
ZmABC044	ZmB84.03G237300.1.v1.2	2286	761	81.70	9.73	17.676
ZmABC045	ZmB84.03G287900.1.v1.2	4437	1478	166.61	8.26	11.240
ZmABC046	ZmB84.03G032000.1.v1.2	4281	1426	161.38	8.16	11.321
ZmABC047	ZmB84.03G170100.1.v1.2	4206	1401	154.53	6.60	-6.994
ZmABC048	ZmB84.03G356100.1.v1.2	3882	1293	139.15	8.08	5.161
ZmABC049	ZmB84.04G022200.1.v1.2	4338	1445	163.38	6.66	-4.213
ZmABC050	ZmB84.04G068500.1.v1.2	3789	1262	137.99	5.86	-14.098
ZmABC051	ZmB84.04G182600.1.v1.2	3789	1262	138.43	9.22	24.465
ZmABC052	ZmB84.04G091600.1.v1.2	1779	592	66.34	6.65	-3.093
ZmABC053	ZmB84.04G112700.1.v1.2	2820	939	104.90	9.34	30.755
ZmABC054	ZmB84.04G210600.1.v1.2	888	295	33.02	6.46	-2.036
ZmABC055	ZmB84.04G264800.1.v1.2	3948	1315	149.22	8.15	10.313
ZmABC056	ZmB84.04G112600.1.v1.2	2871	956	105.34	8.39	11.432
ZmABC057	ZmB84.04G319600.1.v1.2	2853	950	104.35	7.73	3.922
ZmABC058	ZmB84.04G327500.1.v1.2	3732	1243	134.91	8.45	9.256
ZmABC059	ZmB84.04G003400.1.v1.2	2151	716	78.04	8.99	16.088
ZmABC060	ZmB84.04G287000.1.v1.2	1905	634	67.00	9.23	15.638
ZmABC061	ZmB84.04G320300.1.v1.2	4332	1443	162.40	6.70	-3.315
ZmABC062	ZmB84.05G260900.1.v1.2	3744	1247	134.10	7.44	2.973
ZmABC063	ZmB84.05G234700.1.v1.2	2856	951	105.32	6.86	-0.823
ZmABC064	ZmB84.05G126800.1.v1.2	789	262	27.80	7.36	0.683
ZmABC065	ZmB84.05G086400.1.v1.2	2121	706	78.23	8.33	6.849
ZmABC066	ZmB84.05G036600.1.v1.2	1947	648	69.89	7.43	1.493
ZmABC067	ZmB84.05G384900.1.v1.2	3324	1107	120.39	8.37	19.850
ZmABC068	ZmB84.05G235000.1.v1.2	3099	1032	114.46	7.55	4.079
ZmABC069	ZmB84.05G386700.1.v1.2	2163	720	80.43	6.31	-9.052
ZmABC070	ZmB84.06G037800.1.v1.2	2145	714	76.83	9.28	12.443
ZmABC071	ZmB84.06G121300.1.v1.2	2898	965	107.57	8.57	14.93
ZmABC072	ZmB84.06G200100.1.v1.2	2085	694	74.94	8.01	4.333
ZmABC073	ZmB84.06G124900.1.v1.2	4527	1508	169.87	7.14	1.191
ZmABC074	ZmB84.07G029000.1.v1.2	2136	711	77.73	6.33	-5.589
ZmABC075	ZmB84.07G071700.1.v1.2	2112	703	76.13	9.21	15.524
ZmABC076	ZmB84.07G197900.1.v1.2	4062	1353	151.59	8.71	17.881
ZmABC077	ZmB84.07G102200.1.v1.2	2847	948	106.31	8.46	14.468
ZmABC078	ZmB84.07G087300.1.v1.2	3774	1257	141.26	8.27	13.169
ZmABC079	ZmB84.07G072900.1.v1.2	1062	353	38.19	6.61	-2.143
ZmABC080	ZmB84.07G132300.1.v1.2	3891	1296	144.43	6.32	-9.638
ZmABC081	ZmB84.07G057100.1.v1.2	2169	722	78.64	9.01	17.679
ZmABC082	ZmB84.07G087900.1.v1.2	3255	1084	124.07	7.53	4.095
ZmABC083	ZmB84.07G097400.1.v1.2	3813	1270	142.16	6.60	-3.996

ZmABC084	ZmB84.07G132200.1.v1.2	1818	605	68.00	6.79	-0.994
ZmABC085	ZmB84.07G142500.1.v1.2	2211	736	79.72	8.49	7.882
ZmABC086	ZmB84.08G079400.1.v1.2	3468	1155	125.15	7.41	1.934
ZmABC087	ZmB84.08G222200.1.v1.2	3825	1274	136.96	8.26	6.611
ZmABC088	ZmB84.08G198500.1.v1.2	4344	1447	163.21	7.16	1.612
ZmABC089	ZmB84.08G023200.1.v1.2	2004	667	73.05	8.76	8.925
ZmABC090	ZmB84.08G037900.1.v1.2	4539	1512	165.15	6.89	-1.067
ZmABC091	ZmB84.08G058700.1.v1.2	4398	1465	165.17	6.94	-0.544
ZmABC092	ZmB84.08G198400.1.v1.2	4335	1444	162.15	7.34	2.725
ZmABC093	ZmB84.08G178600.1.v1.2	2073	690	75.14	8.98	9.275
ZmABC094	ZmB84.08G295800.1.v1.2	852	283	30.38	9.11	7.769
ZmABC095	ZmB84.08G200400.1.v1.2	1821	606	64.97	9.56	16.081
ZmABC096	ZmB84.08G183500.1.v1.2	3978	1325	148.18	9.13	27.328
ZmABC097	ZmB84.08G069200.1.v1.2	4530	1509	165.73	7.29	3.118
ZmABC098	ZmB84.08G051300.1.v1.2	2289	762	84.36	6.89	-0.444
ZmABC099	ZmB84.08G000700.1.v1.2	3837	1278	137.46	8.49	9.159
ZmABC100	ZmB84.08G079200.1.v1.2	3810	1269	136.04	6.13	-8.715
ZmABC101	ZmB84.08G113600.1.v1.2	528	175	19.15	5.67	-5.895
ZmABC102	ZmB84.08G279500.1.v1.2	2244	747	80.21	9.53	14.507
ZmABC103	ZmB84.08G140300.1.v1.2	2073	690	76.45	9.36	18.240
ZmABC104	ZmB84.09G024400.1.v1.2	4395	1464	163.45	7.39	4.094
ZmABC105	ZmB84.09G183800.1.v1.2	1101	366	40.67	8.79	6.324
ZmABC106	ZmB84.09G097500.1.v1.2	4482	1493	163.52	7.65	5.419
ZmABC107	ZmB84.09G181000.1.v1.2	1815	604	68.20	7.80	4.17
ZmABC108	ZmB84.09G036900.1.v1.2	4443	1480	163.42	7.98	11.408
ZmABC109	ZmB84.09G115900.1.v1.2	1992	663	74.33	9.52	26.572
ZmABC110	ZmB84.09G198600.1.v1.2	4242	1413	155.83	1.18	-15.538
ZmABC111	ZmB84.09G099600.1.v1.2	4509	1502	169.38	8.05	8.280
ZmABC112	ZmB84.09G089000.1.v1.2	3270	1089	119.71	8.74	28.955
ZmABC113	ZmB84.10G124900.1.v1.2	4575	1524	165.75	6.64	-4.883
ZmABC114	ZmB84.10G193900.1.v1.2	4602	1533	171.80	8.10	14.029
ZmABC115	ZmB84.10G218900.1.v1.2	678	225	25.26	10.06	9.568
ZmABC116	ZmB84.10G124000.1.v1.2	3279	1092	121.92	8.58	25.408
ZmABC117	ZmB84.10G224500.1.v1.2	3801	1266	137.41	8.19	7.470
ZmABC118	ZmB84.10G092200.1.v1.2	3783	1260	138.91	8.18	8.545
ZmABC119	ZmB84.10G246600.1.v1.2	2253	750	81.33	9.86	31.939
ZmABC120	ZmB84.10G160600.1.v1.2	3795	1264	137.01	8.37	10.175
ZmABC121	ZmB84.10G168500.1.v1.2	4179	1392	150.10	9.03	25.004
ZmABC122	ZmB84.10G121900.1.v1.2	3810	1269	143.08	6.95	-0.405
ZmABC123	ZmB84.10G058200.1.v1.2	2679	892	99.97	9.22	18.527
ZmABC124	ZmB84.10G035800.1.v1.2	1794	597	66.38	6.52	-4.232
ZmABC125	ZmB84.10G015100.1.v1.2	4428	1475	165.85	8.25	12.389
ZmABC126	ZmB84.10G084600.1.v1.2	2184	727	77.12	9.84	28.392
ZmABC127	ZmB84.K045200.1.v1.2	1857	618	65.89	8.92	11.694

ZmABC128	ZmB84.K048800.1.v1.2	4656	1551	169.99	6.36	-8.611
ZmABC129	ZmB84.K005200.1.v1.2	663	220	24.85	9.85	7.902
ZmABC130	ZmB84.K005700.1.v1.2	519	172	19.68	9.64	4.766
ZmABC131	ZmB84.K004700.1.v1.2	663	220	24.82	9.59	5.905
ZmABC132	ZmB84.K028100.1.v1.2	3543	1180	131.93	7.75	6.702
ZmABC133	ZmB84.K028000.1.v1.2	3669	1222	136.12	6.71	-3.120
